# The nature of sex differences in catecholamine-induced lipolysis in subcutaneous fat cells

**DOI:** 10.1016/j.isci.2025.113988

**Published:** 2025-11-10

**Authors:** Lucas Massier, Daniel P. Andersson, Nathalie Viguerie, Jiawei Zhong, Danae Zareifi, Alastair G. Kerr, Dominique Langin, Peter Arner

**Affiliations:** 1 Helmholtz Institute for Metabolic, Obesity and Vascular Research (HI-MAG) of the Helmholtz Zentrum München at the University of Leipzig and University Hospital Leipzig, Leipzig, Germany; 2 Department of Medicine H7, Karolinska Institutet, Stockholm, Sweden; 3 Department of Endocrinology and Metabolism, Karolinska University Hospital, Stockholm, Sweden; 4 Institute of Metabolic and Cardiovascular Diseases, I2MC, University of Toulouse, Inserm, Toulouse III University - Paul Sabatier (UPS), Toulouse, France; 5 Centre Hospitalier Universitaire de Toulouse, Toulouse, France; 6 Institut Universitaire de France (IUF), Paris, France

**Keywords:** Lipid, Human metabolism, Omics

## Abstract

Women demonstrate a more efficient energy metabolism than men, which is important for sex differences in metabolic health. This dimorphism involves a greater capacity to mobilize lipids from adipose tissue through triglyceride lipolysis following catecholamine stimulation. Herein, we examined the cellular nature of this dimorphism of catecholamine action in human adipocytes from subcutaneous adipose tissue by combining extensive pharmacological experiments with descriptive proteome and transcriptome analyses in large cohorts. We observed two sex-dependent differences in catecholamine-stimulated adipocyte lipolysis: in women, the lipolytic sensitivity (half maximum effective hormone concentration) was 50% decreased, involving increased coupling of antilipolytic alpha-2A adrenoceptors to adenylyl cyclase. However, the maximum lipolytic hormone effect was 50% increased and linked to more efficient mono- and triacylglycerol lipases. Treatment targeting adipocyte lipolysis might be used in men to diminish sex differences in the regulation of lipid metabolism.

## Highlights

• Catecholamine adipocyte lipolysis differs between men and women
• The lipolytic sensitivity is higher in men
• The maximum lipolytic effect is higher in women
• Sex differences stem from α2-adrenoceptor coupling and mono-/triglyceride lipase efficiency

## Graphical abstract

**Figure:**
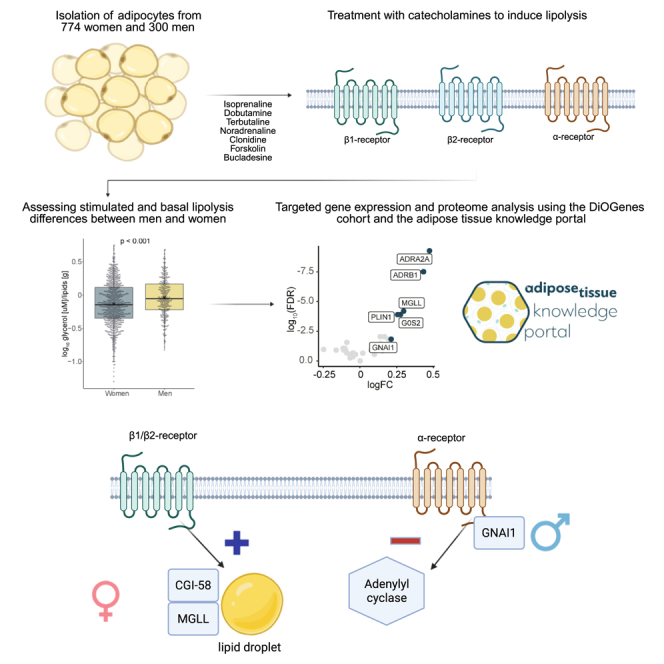

## Introduction

There are fundamental differences between men and women in many aspects of metabolism, which influence physiology as well as pathophysiology of common cardiometabolic conditions such as type 2 diabetes and obesity.^1^ Sex differences in the white adipose tissue capacity to store and mobilize triglycerides are important components in energy metabolism.^2^^,^^3^ This includes the hydrolysis of triglycerides to the end products glycerol and fatty acids in fat cells (lipolysis).^4^

In the regulation of lipolysis in white fat cells, significant species differences exist as reviewed.^5^^,^^6^^,^^7^^,^^8^^,^^9^ Briefly, in humans, only insulin, catecholamines, and natriuretic peptides have pronounced acute effects. In contrast, the natriuretic peptides are ineffective in rodent fat cells.^8^ For catecholamines, a single receptor subtype, the beta-3 adrenoceptor, is involved in the activation of rodent fat cell lipolysis, whereas beta-1 and -2 adrenoceptors are the most important lipolysis activators in human fat cells. In addition, catecholamines exert antilipolytic effects through alpha-2A adrenoceptors (encoded by ADRA2A) in humans; this receptor subtype is not active in rodent fat cells. Finally, DNA fragmentation factor-alpha-like effector A (encoded by CIDEA) modulates lipolysis in humans but is not expressed in mouse white fat cells.

Studies using intravenous hormone infusions^10^^,^^11^ or sympathetic nervous system activation through physical exercise^12^^,^^13^^,^^14^^,^^15^ have established that catecholamine-induced lipolysis is significantly more pronounced in women than in men *in vivo*. However, the cellular and molecular natures of this sex dimorphism are unknown, and studies of fat cells are additionally hampered by the existence of regional differences in the overall and sex-related action of these hormones on lipolysis as reviewed^4^^,^^16^^,^^17^^,^^18^ and detailed in earlier studies.^19^ In one study, omental fat cells had a stronger lipolytic response to catecholamines in men than women.^20^ In three studies, no or minor sex differences in catecholamine-induced lipolysis were found in gluteal fat cells.^21^^,^^22^^,^^23^ These *in vitro* studies cannot explain the sex differences in lipolysis observed *in vivo*. On the other hand, the catecholamine-induced lipolysis in abdominal subcutaneous fat cells is more rapid among women than men,^22^^,^^23^^,^^24^ which is in tune with the above-mentioned *in vivo* studies and reviews.^4^ Quantitatively, lipolysis in abdominal subcutaneous adipose tissue likely has a greater impact on circulating glycerol and fatty acids than lipolysis in omental or gluteal fat, as abdominal (trunk) tissue constitutes approximately 40% of the total adipose mass, making it the largest adipose region.^25^^,^^26^ As all earlier *in vitro* studies used nonselective catecholamines, they provide no mechanistic insight into the sex differences in fat cell lipolysis. Moreover, because previous studies were limited to small groups of women and men (≤30 subjects), it remains unclear whether sex differences in lipolysis are independent of other influencing factors. Additionally, it is unknown whether these differences in catecholamine-induced lipolysis arise at the receptor or post-receptor level.

In order to better understand how sex influences lipolysis, we conducted a large study on white abdominal subcutaneous fat cells incubated with seven selective or nonselective lipolytic and antilipolytic agents acting on the cyclic AMP signal pathway, which is a main mediator of catecholamine-induced lipolysis in human fat cells.^5^^,^^6^^,^^8^^,^^9^^,^^27^ We also measured spontaneous (basal) lipolysis because it is influenced by some regulators associated with the cyclic AMP pathway such as perilipin1 (encoded by PLIN1),^28^ phosphodiesterase 3B (encoded by PDE3B),^29^ and CIDEA.^30^ These pharmacological studies revealed two distinct differences in catecholamine-induced lipolysis localized at specific receptor and post-receptor levels in hormone signaling, which were further identified by targeted gene and protein expression analyses in several large cohorts. We focused on genes in the canonical cyclic AMP signal pathway and included additional regulators that could influence this signaling to lipolysis.

## Results

### Several sex differences in clinical variables

The clinical data for the lipolysis and DiOGenes subjects are shown in Tables S1 and S2, respectively. Well-known differences between men and women were observed. This included higher levels of body mass index (BMI) and body fat as well as lower values for blood pressure, fasting plasma triglycerides, glucose, and total cholesterol, which were found for women compared to men. Women also had higher fasting high-density lipoprotein cholesterol compared to men. In addition, men were older and had smaller fat cells than women in the subjects investigated for lipolysis.

### Increased lipolytic responsiveness and reduced sensitivity to catecholamines in women compared to men

**Figure 1:**
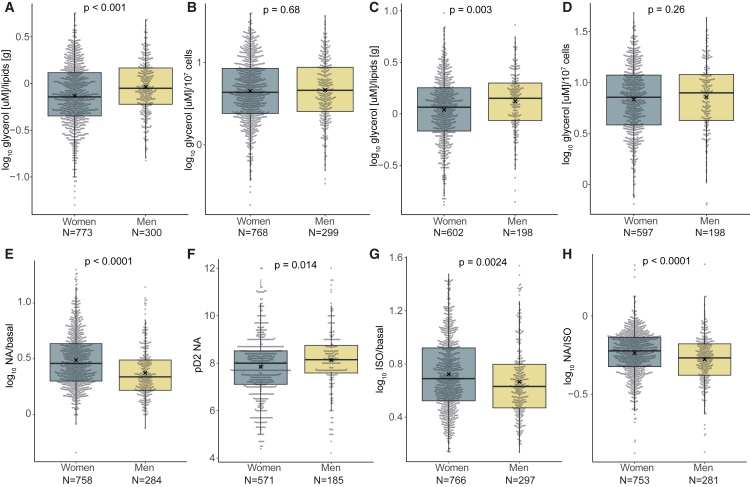
Catecholamine-induced abdominal subcutaneous lipolysis in men and women Lipolysis is measured as release of glycerol from isolated fat cells. It is expressed as (10)log values for all subjects analyzed (A) Basal lipolysis per lipid weight. (B) Basal lipolysis per number of cells. (C and D) Adenosine deaminase-induced lipolysis per lipid weight and (D) per number of cells. (E) Maximum increase of noradrenaline (NA) over basal rate of lipolysis. (F) Half maximum effective lipolytic concentration of NA converted to pD2, as described in STAR Methods. (G and H) (G) Maximum increase of isoprenaline (ISO) over basal lipolysis. (H) Maximum effect of NA divided by maximum effect of ISO. Results are expressed as boxplots with Tukey whiskers and ∗ indicating the mean and compared by unpaired *t* test. *N*, number of subjects. One outlier value in (B and C) was excluded in the graphs and in the statistical analysis.

To better control for sex differences in stimulated lipolysis, we first analyzed lipolysis in the absence of a lipolysis acting agent. Glycerol release per amount of lipids under basal and adenosine deaminase-induced conditions was more rapid among men than women (Figures 1A and 1C). However, there was no sex difference in release when expressed per number of incubated fat cells (Figures 1B and 1D). Next, we analyzed the data for catecholamine-induced lipolysis. For noradrenaline, which measures alpha-2A adrenoceptor antilipolytic and beta adrenoceptor lipolytic effects, responsiveness was increased (high noradrenaline/basal ratio) (Figure 1E**)** but sensitivity was decreased (lower pD2) in women compared to men (Figure 1F**)**. For isoprenaline, which measures lipolytic beta-effects, responsiveness was higher in women than men (Figure 1G**)**. Together, these data suggest a complex sex dimorphism for catecholamine-induced lipolysis with lower sensitivity but increased maximum lipolytic effect in women. To further analyze these differences, we measured the ratio between noradrenaline and isoprenaline responsiveness, which reflects the balance between antilipolytic alpha-2A and lipolytic beta adrenoceptor actions (Figure 1H**)**. The higher the ratio, the less is the alpha effect. Results demonstrated that the ratio was higher in women than men, indicating that the overall sex difference is linked to increased beta-adrenoceptor-mediated lipolytic effects of catecholamines in women.

For the purpose of comparison, the mean values for noradrenaline sensitivity and responsiveness were calculated. The half maximum effective lipolytic concentration, representing lipolytic sensitivity, was almost two times higher in females than in males (13 vs. 7 nmol/L). Maximum ability of noradrenaline to stimulate lipolysis representing responsiveness was about 50% higher in females than in males (fold increase 3.7 and 2.7, respectively).

### Difference in lipolysis is an independent sex dimorphism

**Figure 2:**
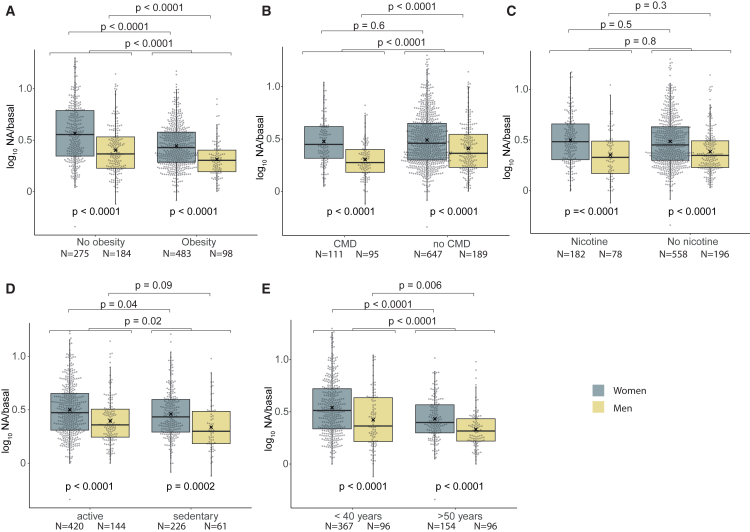
Noradrenaline-induced lipolysis in subgroups of men and women (A) Subjects without or with obesity; the latter was defined as body mass index ≥30 kg/m^2^. (B) Subdivision into having a cardiometabolic disease (CMD) or not, which is defined in STAR Methods. (C) Subdivision into regular use of nicotine or not. (D) Subjects being either physically active or sedentary at leisure and work. (E) Subdivision into being <40 or above 50 years of age at examination. *N*, number of subjects. *p* values below the boxes indicate sex differences within the groups, and *p* values above indicate comparisons across groups for either only female or male subjects or the complete cohort; all tests are performed using Welch’s *t* test, boxplot with Tukey whiskers and ∗ indicating the mean.

Noradrenaline responsiveness was measured in all available subjects and further analyzed in subgroups (Figure 2). The sex dimorphism in lipolysis was maintained in people with or without obesity (Figure 2A) or with or without a cardiometabolic condition (Figure 2B**)**. The same was true for subjects using nicotine or not (Figure 2C**)**, as well as sedentary or physically active subjects (Figure 2D**)**. Finally, subdivision into younger or elderly subjects had no important bearing on the sex dimorphism (Figure 2E**)**. It is observed that values for the elderly were lower than those for younger persons. This is likely because catecholamine-induced lipolysis decreases with aging.^31^

Age, body shape (evaluated by waist-to-hip ratio), fat cell volume, and BMI or body fat could be influenced by sex and/or impact on catecholamine-induced lipolysis. This was investigated by analysis of covariance in Table S3. All these factors were significantly related to variations in noradrenaline-stimulated lipolysis. However, sex still had a significant influence on catecholamine responsiveness. There was no interaction between sex and the co-factors regarding their separate influence on lipolysis. Although men were older than women (Table S2), this factor has apparently no important bearing on the results with lipolysis as judged by the subgroup (Figure 2E**)** and multivariate (Table S3) analyses.

### Sex dimorphism in the action on lipolysis of selective pharmacological agents

**Figure 3:**
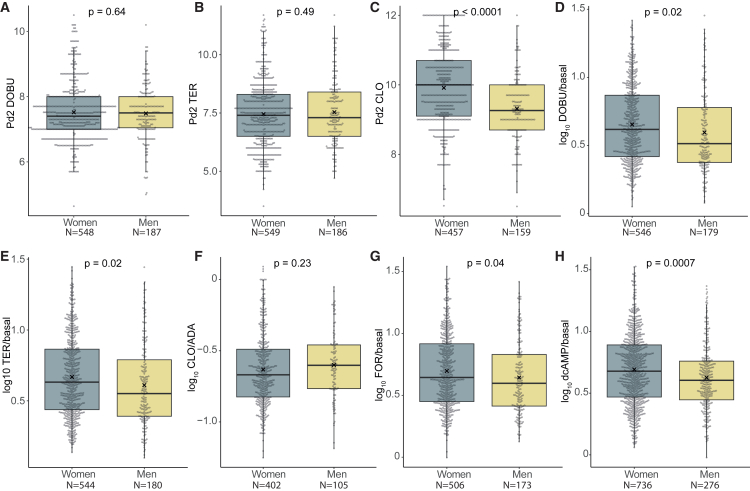
Effect on lipolysis of agents acting at specific steps in the catecholamine-induced lipolysis cascade in the investigated abdominal subcutaneous fat cells DOBU (dobutamine) is a beta-1 adrenoceptor selective agonist used in (A) and D). TER (terbutaline) is a beta-2 adrenoceptor selective agonist used in (B and E). CLO (clonidine) is an alpha-2A selective adrenoceptor agonist used in (C and F). FOR (forskolin) is a selective activator of adenylyl cyclase used in (G). dcAMP (dibutyryl cyclic AMP) is a phosphodiesterase-resistant cyclic adenosine monophosphate analog, which selectively activates the protein kinase A complex and is used in (H). Basal, spontaneous lipolysis; ADA, adenosine deaminase, which selectively breaks down adenosine and is added to basal lipolysis in the clonidine experiments to remove traces of endogenous antilipolytic adenosine. Results are expressed as boxplots with Tukey whiskers where ∗ indicates the mean and compared by unpaired *t* test. *N*, number of subjects.

Next, we performed detailed pharmacological studies with selective lipolytic or antilipolytic agents (Figure 3). Receptor subtype action was investigated by measuring pD2 and responsiveness for alpha-2A and beta-1 and beta-2 adrenoceptors. For dobutamine (Figures 3A and 3D) and terbutaline (Figures 3B and 3E), responsiveness was increased in women, but pD2 was the same in the two sexes. This suggests that dimorphism localized at post-receptor events (below or at adenylyl cyclase) for beta adrenoceptors. For clonidine (Figures 3C and 3F), pD2 was increased in women but responsiveness was the same as in men. This indicates dimorphism at the level of alpha-2A adrenoceptors and/or their coupling to Gi proteins. Furthermore, the pD2 findings with selective agents are in tune with noradrenaline pD2 and imply that decreased lipolytic sensitivity of catecholamines in women is linked to increased activity of alpha-2A adrenoceptor near events. All measured adrenoceptors convey their signaling to lipolysis at the level of adenylyl cyclase. The responsiveness of forskolin (acting at this enzyme level) was higher in women than men (Figure 3G), suggesting that an increased lipolytic action of catecholamines in women is localized at or distal to adenylyl cyclase, i.e., at or beyond the protein kinase A (PKA) complex and/or phosphodiesterase 3B (encoding PDE3B). PKA is stimulated by cyclic AMP, which in turn, can be inactivated by PDE3B-mediated hydrolysis to 5′AMP. The maximum lipolytic action of phosphodiesterase-resistant dibutyryl cyclic AMP was increased in women compared to men (Figure 3H**)**. This excludes phosphodiesterase B as the sole factor responsible for post-adrenoceptor-related sex differences in catecholamine-induced lipolysis between the sexes.

Together with results at basal levels, these findings suggest that a sex dimorphism in PLIN1, PDE3B, or CIDEA might be involved because they influence basal lipolysis.

### Genomics and proteomics analysis supports lipolysis sex dimorphism

**Figure 4:**
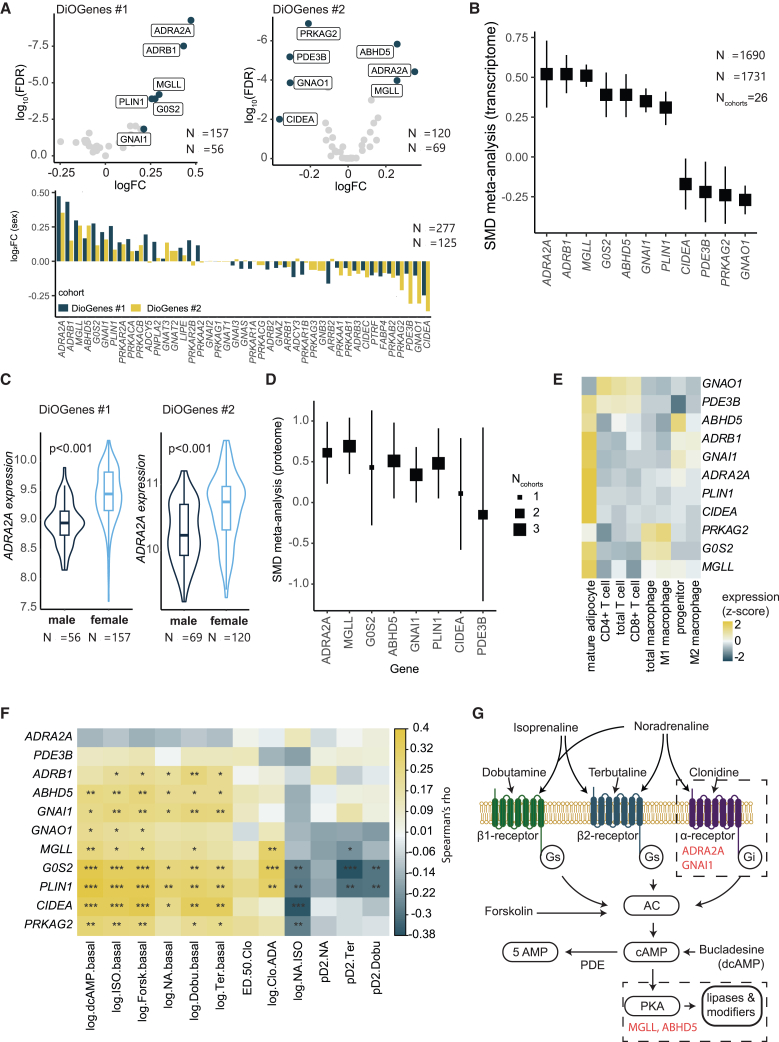
Transcriptional and post-transcriptional analysis of cyclic AMP (cAMP) pathway sex differences (A) The two DiOGenes cohorts were used as discovery cohorts. The 11 genes related to the cAMP pathway showed a differential expression across sexes as shown in individual volcano plots (top), and directionality was largely congruent between the two studies (lower panel). (B and C) Data from the Adipose Tissue Knowledge Portal, which was used to confirm the sex differences across 26 independent cohorts. The strongest sex dimorphism was observed for *ADRA2A*, for which distribution in the DiOGenes cohorts is shown as violin plots in (C). (D) Proteome data from the Adipose Tissue Knowledge Portal, confirming directionality of sex differences on proteome levels. (E) All sex-regulated genes except *GNAO1* are enriched in adipocytes. (F) Expression of sex-regulated genes and their correlation with readouts of stimulated lipolysis as shown in a heatmap. (G) A summary of tested pharmacological inducers of lipolysis, as well as key genes/readouts that are regulated by sex.

To further elucidate the molecular nature of the observed sex differences, we next turned to targeted investigations of subcutaneous adipose gene expression. Forty-three genes in the cyclic AMP signaling pathway or associated with this pathway (Table S4) were analyzed in two discovery cohorts, DiOGenes #1 and #2 (Figure 4A). In women, 7 genes were significantly up- and 4 downregulated in either or both DiOGenes subcohorts as compared with men. Direction of the overall sex effects was congruent for both cohorts. To enhance reproducibility, we validated the sex differences of these 11 genes using the adipose tissue knowledge portal.^32^ For this, we summarized data of 26 different cohorts with available transcriptome data of subcutaneous adipose tissue, including 1,690 women and 1,731 men (Figure 4B**)**. Directionality of the sex dimorphism for all genes could be confirmed. The expression of *ADR2A* (increased in women) showed the strongest sex dimorphism. The distribution of results with this gene according to sex in all examined DiOGenes subjects is shown in Figure 4C.

Proteomics data were available in three portal cohorts (Figure 4D). Of the 11 genes subject to sex dimorphism in mRNA expression, 8 showed detectable protein levels in abdominal subcutaneous adipose tissue, although only 4 (MGLL, ABHD5, GNAI1, and PLIN1) could be detected in all three cohorts. Seven of the latter proteins showed a similar pattern as the corresponding gene expression, six being upregulated and one downregulated in women. CIDEA, which was downregulated for gene expression in women, showed no sex dimorphism on protein level.

The subcellular localization of genes subject to sex dimorphism and the relation between their gene expression in abdominal subcutaneous adipose tissue and lipolysis phenotypes was also investigated. All genes were significantly expressed in fat cells, and many were also expressed in other cell types within the tissue (Figure 4E**)**. All but one, ADR2A, showed a significant correlation between gene expression and stimulation or inhibition of lipolysis (Figure 4F**)**.

### Combining lipolysis and omics

Finally, we combined the outcome of pharmacology, genomics, and proteomics to make a mechanistic model of sex differences in catecholamine-stimulated lipolysis (Figure 4G). Increased expression of adrenoceptor 2A and Gi-1 (*GNAI1*) can in part explain why the lipolytic sensitivity of catecholamines is decreased in women because it may enhance the coupling efficiency of antilipolytic signaling. Increased expression of monoglyceride lipase and the adipose triglyceride cofactor CGI-58 is likely to improve lipase efficiency and partly explain why the maximum lipolytic action of noradrenaline and selective lipolysis stimulators is increased in women. To some degree, decreased expression of phosphodiesterase 3B in women could also be a factor behind increased maximum catecholamine action in this sex, but it is likely to be less important than the aforementioned proteins because of the findings with dibutyryl cyclic AMP and basal lipolysis. For the remaining 6 of the 11 candidates, gene/protein expression and pharmacology data were not in agreement with the pharmacological findings. The upregulation of *ADRB1*, *GOS2*, and *PLIN1* in women does not fit with dobutamine pD2 responsiveness for lipolytic agents or basal lipolysis. The downregulation of *CIDEA*, *PRKAG2*, or *GNAO1* in women does not fit with basal lipolysis, responsiveness of lipolytic agents, or clonidine pD2.

## Discussion

This study provides new insights into sex differences in the regulation of lipolysis in subcutaneous abdominal white fat cells, the body’s largest adipose tissue depot. Our findings reveal a complex sex dimorphism in catecholamine action, highlighting their key role as regulators of lipid mobilization in human adipose tissue.

The pharmacological study points at two hitherto unrecognized sex differences in catecholamine signaling to lipolysis through the cyclic AMP pathway and its connected regulators. First, the lipolytic sensitivity is about 50% lower in women than men, which is likely to be due to increased function of antilipolytic alpha-2A adrenoceptors. Second, the maximum action is ∼50% increased among women, which is probably due to sex differences in post-receptor events at the level of adenylyl cyclase and/or below. However, the pharmacological data indicate that the sex difference in lipolytic stimulation dominates over the antilipolytic effects. The findings with sex seem be independent of numerous cofactors such as obesity, cardiometabolic and physical activity status, nicotine use or age, body shape, fat cell volume, BMI, and total body fat. Many of these factors may by themselves influence lipolysis regulation as reviewed.^5^^,^^9^^,^^27^ This influence could be studied separately using our large cohort but not in this paper focusing on sex differences per se. Other regulators of lipolysis, not examined in this study, may also contribute to sex differences in catecholamine-stimulated lipolysis. These include metabolites like lactate and inflammatory proteins such as tumor necrosis factor-alpha. The present pharmacological data suggest autonomous factors as causative for the sex difference. However, we cannot exclude that variations in hormonal or metabolic milieu are also important for different lipolysis regulation between men and women.

It is unlikely that the differences in lipolysis between men and women are caused by sex hormones. A sex dimorphism was observed in young people, mostly including women with regular menstruations, and in elderly subjects, mainly including women who were menopausal. In addition, previous studies suggest that the menstrual status does not influence catecholamine-induced lipolysis in human abdominal subcutaneous fat cells.^33^ There was also no or little effect on catecholamine-induced lipolysis following gender-affirming treatment in transgender individuals.^34^^,^^35^

The stepwise regulation of lipolysis through the cyclic AMP cascade and the influence of associated lipolysis regulators is complex. For ethical, technical, and practical reasons, it is not possible to remove sufficiently large amounts of adipose tissue in a clinical setting to study all these regulatory events. Therefore, we used targeted gene and protein expression studies of abdominal subcutaneous adipose tissue to obtain additional mechanistic information besides the outcome of the pharmacological investigation. Several pan-genomic studies on sex dimorphism in mRNA expression have been published on human subcutaneous adipose tissue.^36^^,^^37^ These studies highlight sex-specific differences in the expression of genes related to inflammation and mitochondrial function, which may indirectly influence catecholamine-stimulated lipolysis. However, no sex-related differences were observed in the expression of genes within the cyclic AMP signaling pathway. A likely explanation is that previous pan-genomic approaches may have masked such differences due to multiple testing across all genes. To address this, we focused on 43 key genes involved in an extended cyclic AMP signaling pathway (listed in Table S1), analyzing them across multiple cohorts in a meta-analysis using our recently published adipose tissue knowledge portal (adiposetissue.org).^32^ The data in the portal are descriptive. When, however, combined with the pharmacological data, it is possible to deepen the conclusions about the nature behind sex differences in catecholamine-induced lipolysis.

The results section provides detailed insights into the relationship between genomics and lipolysis data. Among the 11 genes exhibiting sex-specific differences in expression, 7 were consistent with the lipolysis findings. All of these genes were enriched in fat cells, and several of them showed a correlation between their expression and activation or inhibition of lipolysis using the different pharmacological agents. Based on pharmacological and omics data, we suggest the following model for sex differences of catecholamine-induced lipolysis in subcutaneous fat cells. The lipolytic sensitivity of noradrenaline is about 50% decreased in women involving increased expression of antilipolytic alpha-2A adrenoceptors and Gi proteins making room for a stronger coupling to adenylyl cyclase. However, the lipolytic, beta adrenoceptor effect of catecholamines is 50% higher in women and men. The latter dominates over the antilipolytic properties of the hormone and relates to increased expression of monoglyceride lipase and the adipose triglyceride cofactor CGI-58. Lower expression of phosphodiesterase 3 in women may, to a minor extent, contribute to their increased catecholamine-induced lipolysis compared with men. It is also possible that post-translational factors related to cyclic AMP signaling and other factors such as inflammation and mitochondrial function not examined herein could be of importance for sex dimorphism in lipolysis. It should be stressed that our conclusions are based on pharmacology and descriptive omics. A true mechanistic explanation can only be obtained by direct manipulation of the genes/factors involved in the sex differences regarding lipolysis regulation.

Sex differences should be considered in metabolic regulation.^2^ Because physical exercise improves catecholamine-stimulated lipolysis,^38^ it might be beneficial to use exercise programs in men to diminish the sex differences in the control of lipid metabolism. Additionally, it could be an advantage to block the antilipolytic properties of catecholamines in women. Clearly, these hypotheses must be verified by direct studies.

Sex differences in lipolysis regulation are not only found for catecholamines but also include the effect of insulin.^39^ Women have a more pronounced antilipolytic effect of insulin than men, but this sex dimorphism is only observed when obesity is present.

### Limitations of the study

There are some caveats with this study. It is not population based. However, the risk of bias in selection of study subjects is minimized by investigations of large groups recruited in different ways and correction of lipolysis data for numerous confounding factors. We only investigated abdominal subcutaneous adipose tissue. However, it is by far the body's largest adipose depot and has probably a more prominent influence on overall lipolysis than other adipose regions. In the lipolysis and DiOGenes cohorts, more women than men were recruited. On the other hand, the statistical power calculation suggested that we could detect minor differences between small-sized groups.

In summary, the sex differences in catecholamine-induced subcutaneous fat cell lipolysis are complex. The lipolytic hormone sensitivity is decreased in women due to more efficient coupling of antilipolytic alpha-2A adrenoceptors to adenylyl cyclase compared to males, but the maximum lipolytic effect is increased in women because of the enhanced ability of beta adrenoceptors to activate lipolysis involving more effective lipases in this sex. The interpretations are related to the analysis of pharmacological and omics data. A definitive mechanistic conclusion about sex differences in lipolysis necessitates direct examination of genes/proteins involved in the dimorphism.

## Resource availability

### Lead contact

Requests for further information and resources should be directed to and will be fulfilled by the lead contact, Peter Arner (peter.arner@ki.se).

### Materials availability

This study did not generate new unique reagents.

### Data and code availability

• Data: This paper analyses existing, publicly available data (through the adipose tissue knowledge portal). Included datasets are detailed in the key resources table with their respective repository (GEO, EBI-EMBL, and PRIDE) accession number, adapted from https://github.com/MassierLab/ATportal_manuscript/blob/main/Manuscript/Table1_cohort.md.
• Code: No new original code was generated for this paper
• Other: Additionally, clinical data related to lipolysis reported in this paper will be shared by the lead contact upon reasonable request. Any additional information required to reanalyze the data reported in this paper is available from the lead contact upon request.

## Acknowledgments

We thank the participants and research staffs of Karolinska Institutet and DiOGenes for their valuable contributions. We thank Professor Jyrki Kukkonen, University of Helsinki (Helsinki, Finland), for valuable discussions about G-protein actions. The study was funded by grants from the 10.13039/100009481Stockholm County Council (D.P.A.), the Strategic Program for Diabetes Research and Center for 10.13039/501100018713Innovative Medicine at 10.13039/501100004047Karolinska Institutet (D.P.A.), the 10.13039/501100018846Swedish Society of Medicine (D.P.A.), the Swedish Research Council (L.M.), the European Association for the Study of Diabetes (L.M.), and the German Diabetes Association (L.M.). The funders had no role in the study design, conduct, collection, management, analysis, interpretation of data, writing or reviewing the manuscript, or decision to submit the manuscript for publication.

## Author contributions

Conceptualization, L.M., D.P.A., and P.A.; methodology, L.M., D.P.A., and P.A.; investigation, L.M., D.P.A., A.G.K., and P.A.; writing – original draft, L.M. and P.A.; writing – review & editing, L.M., D.P.A., and P.A.; funding acquisition, L.M., D.P.A., and P.A.; resources, L.M., J.Z., D.Z., D.L., N.V., and P.A.; supervision, L.M. and P.A.

## Declaration of interests

The authors declare no competing interests.

## STAR★Methods

### Key resources table

**Table:** 

REAGENT or RESOURCE	SOURCE	IDENTIFIER
**Chemicals, peptides, and recombinant proteins**		

Citanest	Aspen Nordic	N01BB04
Collagenase from Clostridium histolyticum type I	Sigma-Aldrich	C0130
Bovine serum albumin fraction V	Sigma-Aldrich	BSAV-RO
Adenosine deaminase	Sigma-Aldrich	9026-93-1
Noradrenaline	Sigma-Aldrich	108341-18-0
Isoprenaline hydrochloride	Sigma-Aldrich	51-30-9
Forskolin	Selleck Chemicals	S2449
Dobutamine hydrochloride	Sigma-Aldrich	49745-95-1
Terbutaline	Sigma-Aldrich	23031-32-5
Clonidine hydrochloride	Sigma-Aldrich	4205-91-8
Dibutyryl cyclic AMP	Selleck Chemicals	S7858
Glycerol Kinase	Sigma-Aldrich	9030-66-4
Firefly luciferase	LKB Wallac	N\A
Luciferin	LKB Wallac	N\A
Magnesium acetate	LKB Wallac	N\A

**Deposited data**		

Kerr^40^	32406570	GSE199063
Imbert et al.^41^	34415992	GSE141221
Armenise et al.^42^	28793995	GSE95640
Arner^43^	27535281	GSE76399
Krieg^44^	34598978	communication with author
Keller^45^	28123940	communication with author
Petrus^46^	30332637	GSE59034
Arner^47^	22688341	GSE25402
Arner^48^	29861390	GSE113080
Nono Nankam^49^	32581226	communication with author
Civelek^50^	28257690	GSE70353
Raulerson^51^	31564431	GSE135134
Stančáková^52^	22553379	GSE32512
Winnier^53^	25830378	GSE64567
Nookaew^54^	23264395	GSE27916
Das^55^	25868721	GSE65221
Barberio^56^	31798691	GSE88837
Vink^57^	27840413	GSE77962
Sharma^58^	26789776	GSE95674
GTEx microarray 2013	N\A	GSE45878
Lonsdale^59^	23715323	gtexportal
Aguet^60^	29022597	gtexportal
Aguet^61^	32913098	gtexportal
Drong^62^	23431366	E-MTAB-54
Naukkarinen^63^	24100782	E-MTAB-1895
Defour^64^	32866087	GSE154610
Johansson^65^	22648723	GSE35411
Matualatupauw^66^	28529330	GSE87382
Du Plessis^67^	26028579	GSE58979
Van Bussel^68^	28337029	GSE84046
MacLaren^69^	20105310	GSE15524
Hardy^70^	20678967	GSE20950
Salcedo-Tacuma^71^	35715414	GSE188799
Aguilera^72^	25856673	GSE9624
Grundberg^73^	22941192	E-TABM-1140
Heinonen^74^	27734103	GSE92405
Bollepalli^75^	28978976	GSE103766
Rey^76^	33671464	GSE166047
Diamanti^77^	36198307	PXD027597
Zhong^32^	39983713	PXD057443
Hruska^78^	37792298	PXD041721
Krieg^44^	34598978	communication with author

**Software and algorithms**		

Adipose tissue knowledge portal	https://adiposetissue.org/	N\A
Statview v5.0	SAS Institute Inc.	N\A
R v4.4.2	R Core Team	N\A

**Other**		

Luminometer	LKB Wallac	Typ 1251

### Experimental model and study participants details

#### Subjects

From 1987 to 2020, 774 women and 300 men (adults living around Stockholm, Sweden) were included to investigate catecholamine induced abdominal subcutaneous fat cell lipolysis. They have previously been investigated in other type of studies as exemplified.^48^^,^^79^^,^^80^ The subjects were recruited by local advertising and/or from the hospital's departments of Medicine or Surgery. They self-reported to be in general good health. However, according to a health questionnaire 208 were treated for cardiometabolic disease (hypertension, hyperlipidemia and/or type 2 diabetes). About 5% were of non-European origin. In general, all subject who were willing to participate were included, expect for cases with acute ongoing severe disease. Sex was determined at birth and confirmed at examination. The participants were investigated in the morning in the overnight fasting state. All subjects were body weight stable for at least 3 months according to self-report (<±2 kg change). Height, body weight, circumferences of waist and hip, plus body fat (by impedance) were determined, which was followed by venous blood sampling for routine clinical chemistry measures as described.^81^ Abdominal subcutaneous adipose tissue was obtained by needle aspiration from the periumbilical area using Citanest (AspenNordic, Ballerup, Denmark) as local anesthesia agent. It contains 5 mg/mL of prilocaine but no catecholamine. Physical activity was assessed by a four-graded scale. These scores have been validated and are highly specific for classification into a sedentary (score 1) or active (score 2 or more) phenotype.^82^ Obesity was defined as body mass index (BMI) ≥30 kg/m^2^. A second adult group of 277 women and 125 men were included solely for gene expression analysis. They participated in a pan-European, multi-centre, randomized controlled dietary intervention program termed Diogenes with trial number NCT00390637.^83^ Herein, we used data on abdominal subcutaneous adipose gene expression and clinical data from the baseline examination. Results were additionally validated in 1731 female and 1690 male subjects on transcript and 89 female and 82 male subjects on proteome level using data obtained through the adipose tissue knowledge portal (adiposetissue.org).^32^ The lipolysis data collection is based on several previous projects, and all have been approved by the Regional Ethics Committee in Stockholm, Sweden under the following ethics permit numbers: diary numbers 2018 809-31, 2022-03062-02 and 2025-03186-02. An ethics permit from 2018 (Diary number 2018/809-31) allowed us to retrospectively analyze all clinical and adipose data. A third group of 132 adult women^84^ were retrospectively studied for relationship between abdominal subcutaneous adipose gene expression and lipolysis phenotypes The different studies for lipolysis were explained in detail to each participant and informed written consent was obtained. Diogenes studies were performed according to the latest version of the Declaration of Helsinki. Local ethics committees at the different investigation sites in Europe approved all procedures and written informed consent was obtained from all participants.

### Method details

#### Fat cell lipolysis

Methods to study human fat cell metabolism in small adipose tissue samples that could be obtained in an ambulatory setting were previously described in detail.^85^ Briefly, with the help of the same four laboratory technicians throughout the study, collagenase isolated fat cells were prepared, and one portion was used for determination of mean fat cell volume as described.^86^ Diluted fat cell suspensions were incubated in duplicate in the absence (basal) or presence of a lipolysis acting agent for 2 h at 37°C in a buffer (pH 7.4) containing glucose, albumin, and, in some cases (see below), adenosine deaminase (ADA, 1mU/l) to remove adenosine, which inhibits lipolysis. Glycerol in the medium was measured as lipolysis index with an ultrasensitive bioluminescence method^86^ and the concentration increased in a linear fashion with the incubation time for at least 4. The following agents were, depended on availability of biomaterials, added at 3 to 12 different concentrations (ranging from 1 pmol/L to 1 mmol/L dependent on the agent used) to the incubation medium: noradrenaline (natural catecholamine acting on all adrenoceptor subtypes), isoprenaline (nonselective lipolytic beta-adrenoceptor agonist), dobutamine (selective lipolytic beta-1 adrenoceptor agonist), terbutaline (selective lipolytic beta-2 adrenoceptor agonist), clonidine (selective antilipolytic alpha-2 adrenoceptor agonist) in the presence or absence of ADA, forskolin (selective lipolytic activator of adenylyl cyclase) or dibutyryl cyclic AMP (phosphodiesterase resistant lipolytic cyclic AMP analogue). There is no consensus on how to express absolute rates of lipolysis. Herein, we expressed basal, or ADA induced lipolysis as glycerol release per amount of incubated lipids or number of fat cells. Lipolysis induced by the different agents was expressed as a ratio over basal or ADA glycerol release to avoid the influence of amounts of lipids or cells in the incubate. The effect was calculated at the maximum effective concentration (responsiveness) for all agents. Catecholamine and adrenoceptor sensitivities were determined by measuring the half maximum effective noradrenaline or receptor selective agonist concentration from the concentration-response curves. This value was transformed to the negative 10 log molar value (pD2). Beta- and alpha adrenoceptors are spare receptors for human fat cell lipolysis.^87^ Therefore, responsiveness and pD2 reflect receptor distal and near events, respectively.^88^ We could not always make complete lipolysis experiments using all concentrations of each of the pharmacological substances. In such cases, single maximum effective concentrations of the different agents were used.

#### Selection of genes for possible link to lipolysis

We conducted a targeted analysis of genes in the cyclic AMP signaling pathway, as well as genes outside the pathway that could influence its various steps, using a comprehensive selection that included genes potentially involved even in the absence of direct lipolysis data. We also included the genes encoding adenosine monophosphate-activated protein kinase complex (AMPK), because they may act as breaks of catecholamine induced lipolysis by interfering with the lipases.^89^ A list of the 43 genes and description of their possible effect on lipolysis are found in Table S1. Details of the genes can be found elsewhere.^5^^,^^6^^,^^7^^,^^9^^,^^27^^,^^28^^,^^30^^,^^90^^,^^91^^,^^92^^,^^93^

#### Genomics and proteomics

Details on the DiOGenes cohorts were previously published.^41^^,^^42^ Briefly, for RNAseq total RNA was extracted, quantified and quality controlled. Gene expression was then examined either by using 100-nucleotide long paired-endRNA sequencing with an Illumina HiSeq 2500 of libraries prepared using the QuantSeq 3′ mRNA-Seq Library Prep Kit from Lexogen and SBS v4 chemistry (DiOGenes #1^41^) or Illumina HiSeq 2000 sequencing of libraries prepared by using the Illumina TruSeq kit following the manufacturer’s standard protocols (DiOGenes #2^42^). Sequencing was performed for samples having both baseline and after-treatment investigation but herein only the former samples were used. Demultiplexing was carried out with Casava (http://support.illumina.com/sequencing/sequencing_software/casava.html); the resulting FASTQ files were then mapped onto the human genome (GRCh37 assembly) with RNA-STAR^94^ with the use of default parameters. Sequencing quality was evaluated by using FastQC (https://www.bioinformatics.babraham.ac.uk/projects/fastqc/). Mapping quality was assessed by using Rsamtools (https://bioconductor.org/packages/release/bioc/html/Rsamtools.html). The number of reads mapping onto genes was retrieved by using GenomicAlignments.^95^ Annotation was performed by using 64,102 genes from the GRCh37.75 assembly generated with the use of the AnnotationDbi R package (https://bioconductor.org/packages/release/bioc/html/AnnotationDbi.html). The values for mRNA are presented as log 2 transformed relative expression. In the present study we selected genes involved regulating the early and late steps of catecholamine induced signaling through the cyclic AMP pathway.^5^^,^^6^^,^^7^^,^^8^ For the validation of functional importance for lipolysis of hit genes we used data from a recently published study of abdominal subcutaneous adipose tissue of 132 women^84^ where pan genomic gene expression by Affymetrix microarray and lipolysis in isolated fat cells were available.

### Quantification and statistical analysis

Analyses of lipolysis and clinical data were performed in Statview 5.0 (SAS, Institute Inc., Buckinghamshire, UK) and R v4.4.2. Analysis of omics data is described in the section above. Values for lipolysis parameters in fat cells (primary endpoints) were normalized by (10)log transformation. Outliers were defined as values with absolute z-transformed values over 3 standard deviations. Two such cases were found and removed from analysis. Clinical and lipolysis results were given as mean and (range) in tables and as boxplots with Tukey whiskers. Meta-analysis of gene expression was presented according to guidelines,^96^ and meta standard mean differences and standard deviation thereof were plotted. The primary comparator was sex, which was analyzed by unpaired *t* test or Fisher's exact test. When several factors were compared for relation with lipolysis, we used one way analysis of covariance (ANCOVA) to analyze their individual contribution as well as their interaction. Correlations were calculated using Spearmańs rho and adjusted for multiple testing. Besides sex, we included the following co-factors: BMI or % body fat, waist-to-hip ratio, mean fat cell volume and age which were not influenced by each other in an important way. Subgroup analyses were made comparing noradrenaline induced lipolysis between men and women in conditions which might influence lipolysis. Those were subjects having obesity or not, being sedentary or physically active, using nicotine or not, having a cardiometabolic disease or not and different age groups. In the latter case we investigated those <40 years or >50 years of age to indirectly evaluate pre- and post-menstrual status in women. For all comparisons a two-tailed test was used and *p* < 0.05 was defined as statistically significant. Prior to termination of inclusion of subjects in the lipolysis study we made a power calculation using previously recorded mean ± SD values for (10)log noradrenaline/basal lipolysis in 36 body weight stabile women, which was 0.53 ± 0.2.^48^ In two groups of equal size, we could detect a 0.14 difference in lipolysis (small effect size) between the sexes in 60 subjects of each group with 80% power and at *p* = 0.05 using two-sided *t* test. As the number of women was larger than of men the statistical power calculation suggests that we had adequate statistical power in the present study to study lipolysis differences small subgroups. The mode of expression and statistical analysis of the gene/protein expression data are given in the section above.

### Additional resources

For transcriptome and proteome analysis, we utilized the processed datasets collected for, as well as summary statistics obtained through, the adipose tissue knowledge portal (adiposetissue.org).^84^^,^^85^ All included datasets are summarized in Table S5. For primary analysis, we studied the DiOGenes cohorts, which were previously published and extensively described.^84^^,^^85^

## References

[1] Clegg D.J., Mauvais-Jarvis F. (2018). An integrated view of sex differences in metabolic physiology and disease. Mol. Metab..

[2] Wu B.N., O’Sullivan A.J. (2011). Sex differences in energy metabolism need to be considered with lifestyle modifications in humans. J. Nutr. Metab..

[3] Goossens G.H., Jocken J.W.E., Blaak E.E. (2021). Sexual dimorphism in cardiometabolic health: the role of adipose tissue, muscle and liver. Nat. Rev. Endocrinol..

[4] Jensen M.D. (1997). Lipolysis: contribution from regional fat. Annu. Rev. Nutr..

[5] Frühbeck G., Méndez-Giménez L., Fernández-Formoso J.-A., Fernández S., Rodríguez A. (2014). Regulation of adipocyte lipolysis. Nutr. Res. Rev..

[6] Grabner G.F., Xie H., Schweiger M., Zechner R. (2021). Lipolysis: cellular mechanisms for lipid mobilization from fat stores. Nat. Metab..

[7] Li Y., Li Z., Ngandiri D.A., Llerins Perez M., Wolf A., Wang Y. (2022). The Molecular Brakes of Adipose Tissue Lipolysis. Front. Physiol..

[8] Morigny P., Boucher J., Arner P., Langin D. (2021). Lipid and glucose metabolism in white adipocytes: pathways, dysfunction and therapeutics. Nat. Rev. Endocrinol..

[9] Yang A., Mottillo E.P. (2020). Adipocyte lipolysis: from molecular mechanisms of regulation to disease and therapeutics. Biochem. J..

[10] Horton T.J., Dow S., Armstrong M., Donahoo W.T. (2009). Greater systemic lipolysis in women compared with men during moderate-dose infusion of epinephrine and/or norepinephrine. J. Appl. Physiol..

[11] Schmidt S.L., Bessesen D.H., Stotz S., Peelor F.F., Miller B.F., Horton T.J. (2014). Adrenergic control of lipolysis in women compared with men. J. Appl. Physiol..

[12] Arner P., Kriegholm E., Engfeldt P., Bolinder J. (1990). Adrenergic regulation of lipolysis in situ at rest and during exercise. J. Clin. Investig..

[13] Hellström L., Blaak E., Hagström-Toft E. (1996). Gender differences in adrenergic regulation of lipid mobilization during exercise. Int. J. Sports Med..

[14] Horton T.J., Pagliassotti M.J., Hobbs K., Hill J.O. (1998). Fuel metabolism in men and women during and after long-duration exercise. J. Appl. Physiol..

[15] Moro C., Pillard F., de Glisezinski I., Crampes F., Thalamas C., Harant I., Marques M.-A., Lafontan M., Berlan M. (2007). Sex differences in lipolysis-regulating mechanisms in overweight subjects: effect of exercise intensity. Obes. Silver Spring Md.

[16] Karastergiou K., Smith S.R., Greenberg A.S., Fried S.K. (2012). Sex differences in human adipose tissues - the biology of pear shape. Biol. Sex Differ..

[17] White U.A., Tchoukalova Y.D. (2014). Sex dimorphism and depot differences in adipose tissue function. Biochim. Biophys. Acta.

[18] Gavin K.M., Bessesen D.H. (2020). Sex Differences in Adipose Tissue Function. Endocrinol. Metab. Clin. North Am..

[19] Ostman J., Arner P., Engfeldt P., Kager L. (1979). Regional differences in the control of lipolysis in human adipose tissue. Metabolism.

[20] Lönnqvist F., Thörne A., Large V., Arner P. (1997). Sex differences in visceral fat lipolysis and metabolic complications of obesity. Arterioscler. Thromb. Vasc. Biol..

[21] Richelsen B. (1986). Increased alpha 2- but similar beta-adrenergic receptor activities in subcutaneous gluteal adipocytes from females compared with males. Eur. J. Clin. Invest..

[22] Leibel R.L., Hirsch J. (1987). Site- and sex-related differences in adrenoreceptor status of human adipose tissue. J. Clin. Endocrinol. Metab..

[23] Wahrenberg H., Lönnqvist F., Arner P. (1989). Mechanisms underlying regional differences in lipolysis in human adipose tissue. J. Clin. Investig..

[24] Crampes F., Riviere D., Beauville M., Marceron M., Garrigues M. (1989). Lipolytic response of adipocytes to epinephrine in sedentary and exercise-trained subjects: sex-related differences. Eur. J. Appl. Physiol..

[25] Yim J.-E., Heshka S., Albu J.B., Heymsfield S., Gallagher D. (2008). Femoral-gluteal subcutaneous and intermuscular adipose tissues have independent and opposing relationships with CVD risk. J. Appl. Physiol..

[26] Karpe F., Vasan S.K., Humphreys S.M., Miller J., Cheeseman J., Dennis A.L., Neville M.J. (2018). Cohort Profile: The Oxford Biobank. Int. J. Epidemiol..

[27] Arner P., Langin D. (2014). Lipolysis in lipid turnover, cancer cachexia, and obesity-induced insulin resistance. Trends Endocrinol. Metab..

[28] Desgrouas C., Thalheim T., Cerino M., Badens C., Bonello-Palot N. (2024). Perilipin 1: a systematic review on its functions on lipid metabolism and atherosclerosis in mice and humans. Cardiovasc. Res..

[29] Degerman E., Ahmad F., Chung Y.W., Guirguis E., Omar B., Stenson L., Manganiello V. (2011). From PDE3B to the regulation of energy homeostasis. Curr. Opin. Pharmacol..

[30] Nordström E.A., Rydén M., Backlund E.C., Dahlman I., Kaaman M., Blomqvist L., Cannon B., Nedergaard J., Arner P. (2005). A human-specific role of cell death-inducing DFFA (DNA fragmentation factor-alpha)-like effector A (CIDEA) in adipocyte lipolysis and obesity. Diabetes.

[31] Gao H., Arner P., Beauchef G., Guéré C., Vie K., Dahlman I., Mejhert N., Rydén M. (2020). Age-Induced Reduction in Human Lipolysis: A Potential Role for Adipocyte Noradrenaline Degradation. Cell Metab..

[32] Zhong J., Zareifi D., Weinbrenner S., Hansen M., Klingelhuber F., Nono Nankam P.A., Frendo-Cumbo S., Bhalla N., Cordeddu L., de Castro Barbosa T. (2025). adiposetissue.org: A knowledge portal integrating clinical and experimental data from human adipose tissue. Cell Metab..

[33] Rydén M., Gao H., Arner P. (2020). Influence of Aging and Menstrual Status on Subcutaneous Fat Cell Lipolysis. J. Clin. Endocrinol. Metab..

[34] Elbers J.M., de Jong S., Teerlink T., Asscheman H., Seidell J.C., Gooren L.J. (1999). Changes in fat cell size and in vitro lipolytic activity of abdominal and gluteal adipocytes after a one-year cross-sex hormone administration in transsexuals. Metabolism.

[35] Subramanian N., Wiik A., Rullman E., Melin M., Lundberg T.R., Flanagan J., Holmberg M., Dekanski A., Dhejne C., Arver S. (2024). Adipokine secretion and lipolysis following gender-affirming treatment in transgender individuals. J. Endocrinol. Investig..

[36] Anderson W.D., Soh J.Y., Innis S.E., Dimanche A., Ma L., Langefeld C.D., Comeau M.E., Das S.K., Schadt E.E., Björkegren J.L.M. (2020). Sex differences in human adipose tissue gene expression and genetic regulation involve adipogenesis. Genome Res..

[37] Rey F., Messa L., Pandini C., Maghraby E., Barzaghini B., Garofalo M., Micheletto G., Raimondi M.T., Bertoli S., Cereda C. (2021). RNA-seq Characterization of Sex-Differences in Adipose Tissue of Obesity Affected Patients: Computational Analysis of Differentially Expressed Coding and Non-Coding RNAs. J. Pers. Med..

[38] Thompson D., Karpe F., Lafontan M., Frayn K. (2012). Physical activity and exercise in the regulation of human adipose tissue physiology. Physiol. Rev..

[39] Arner P., Viguerie N., Massier L., Rydén M., Astrup A., Blaak E., Langin D., Andersson D.P. (2024). Sex differences in adipose insulin resistance are linked to obesity, lipolysis and insulin receptor substrate 1. Int. J. Obes..

[40] Kerr A.G., Andersson D.P., Rydén M., Arner P., Dahlman I. (2020). Long-term changes in adipose tissue gene expression following bariatric surgery. J. Intern. Med..

[41] Imbert A., Vialaneix N., Marquis J., Vion J., Charpagne A., Metairon S., Laurens C., Moro C., Boulet N., Walter O. (2022). Network Analyses Reveal Negative Link Between Changes in Adipose Tissue GDF15 and BMI During Dietary-induced Weight Loss. J. Clin. Endocrinol. Metab..

[42] Armenise C., Lefebvre G., Carayol J., Bonnel S., Bolton J., Di Cara A., Gheldof N., Descombes P., Langin D., Saris W.H. (2017). Transcriptome profiling from adipose tissue during a low-calorie diet reveals predictors of weight and glycemic outcomes in obese, nondiabetic subjects. Am. J. Clin. Nutr..

[43] Arner P., Sahlqvist A.-S., Sinha I., Xu H., Yao X., Waterworth D., Rajpal D., Loomis A.K., Freudenberg J.M., Johnson T. (2016). The epigenetic signature of systemic insulin resistance in obese women. Diabetologia.

[44] Krieg L., Didt K., Karkossa I., Bernhart S.H., Kehr S., Subramanian N., Lindhorst A., Schaudinn A., Tabei S., Keller M. (2022). Multiomics reveal unique signatures of human epiploic adipose tissue related to systemic insulin resistance. Gut.

[45] Keller M., Hopp L., Liu X., Wohland T., Rohde K., Cancello R., Klös M., Bacos K., Kern M., Eichelmann F. (2017). Genome-wide DNA promoter methylation and transcriptome analysis in human adipose tissue unravels novel candidate genes for obesity. Mol. Metab..

[46] Petrus P., Mejhert N., Corrales P., Lecoutre S., Li Q., Maldonado E., Kulyté A., Lopez Y., Campbell M., Acosta J.R. (2018). Transforming Growth Factor-β3 Regulates Adipocyte Number in Subcutaneous White Adipose Tissue. Cell Rep..

[47] Arner E., Mejhert N., Kulyté A., Balwierz P.J., Pachkov M., Cormont M., Lorente-Cebrián S., Ehrlund A., Laurencikiene J., Hedén P. (2012). Adipose tissue microRNAs as regulators of CCL2 production in human obesity. Diabetes.

[48] Arner P., Andersson D.P., Bäckdahl J., Dahlman I., Rydén M. (2018). Weight Gain and Impaired Glucose Metabolism in Women Are Predicted by Inefficient Subcutaneous Fat Cell Lipolysis. Cell Metab..

[49] Nono Nankam P.A., Blüher M., Kehr S., Klöting N., Krohn K., Adams K., Stadler P.F., Mendham A.E., Goedecke J.H. (2020). Distinct abdominal and gluteal adipose tissue transcriptome signatures are altered by exercise training in African women with obesity. Sci. Rep..

[50] Civelek M., Wu Y., Pan C., Raulerson C.K., Ko A., He A., Tilford C., Saleem N.K., Stančáková A., Scott L.J. (2017). Genetic Regulation of Adipose Gene Expression and Cardio-Metabolic Traits. Am. J. Hum. Genet..

[51] Raulerson C.K., Ko A., Kidd J.C., Currin K.W., Brotman S.M., Cannon M.E., Wu Y., Spracklen C.N., Jackson A.U., Stringham H.M. (2019). Adipose Tissue Gene Expression Associations Reveal Hundreds of Candidate Genes for Cardiometabolic Traits. Am. J. Hum. Genet..

[52] Stancáková A., Civelek M., Saleem N.K., Soininen P., Kangas A.J., Cederberg H., Paananen J., Pihlajamäki J., Bonnycastle L.L., Morken M.A. (2012). Hyperglycemia and a common variant of GCKR are associated with the levels of eight amino acids in 9,369 Finnish men. Diabetes.

[53] Winnier D.A., Fourcaudot M., Norton L., Abdul-Ghani M.A., Hu S.L., Farook V.S., Coletta D.K., Kumar S., Puppala S., Chittoor G. (2015). Transcriptomic identification of ADH1B as a novel candidate gene for obesity and insulin resistance in human adipose tissue in Mexican Americans from the Veterans Administration Genetic Epidemiology Study (VAGES). PloS One.

[54] Nookaew I., Svensson P.-A., Jacobson P., Jernås M., Taube M., Larsson I., Andersson-Assarsson J.C., Sjöström L., Froguel P., Walley A. (2013). Adipose tissue resting energy expenditure and expression of genes involved in mitochondrial function are higher in women than in men. J. Clin. Endocrinol. Metab..

[55] Das S.K., Ma L., Sharma N.K. (2015). Adipose tissue gene expression and metabolic health of obese adults. Int. J. Obes..

[56] Barberio M.D., Nadler E.P., Sevilla S., Lu R., Harmon B., Hubal M.J. (2019). Comparison of visceral adipose tissue DNA methylation and gene expression profiles in female adolescents with obesity. Diabetol. Metab. Syndr..

[57] Vink R.G., Roumans N.J., Fazelzadeh P., Tareen S.H.K., Boekschoten M.V., van Baak M.A., Mariman E.C. (2017). Adipose tissue gene expression is differentially regulated with different rates of weight loss in overweight and obese humans. Int. J. Obes..

[58] Sharma N.K., Sajuthi S.P., Chou J.W., Calles-Escandon J., Demons J., Rogers S., Ma L., Palmer N.D., McWilliams D.R., Beal J. (2016). Tissue-Specific and Genetic Regulation of Insulin Sensitivity-Associated Transcripts in African Americans. J. Clin. Endocrinol. Metab..

[59] Lonsdale J., Thomas J., Salvatore M., Phillips R., Lo E., Shad S., Hasz R., Walters G., Garcia F., Young N. (2013). The Genotype-Tissue Expression (GTEx) project. Nat. Genet..

[60] GTEx Consortium (2017). Genetic effects on gene expression across human tissues. Nature.

[61] GTEx Consortium (2020). The GTEx Consortium atlas of genetic regulatory effects across human tissues. Science.

[62] Drong A.W., Nicholson G., Hedman A.K., Meduri E., Grundberg E., Small K.S., Shin S.-Y., Bell J.T., Karpe F., Soranzo N. (2013). The presence of methylation quantitative trait loci indicates a direct genetic influence on the level of DNA methylation in adipose tissue. PloS One.

[63] Naukkarinen J., Heinonen S., Hakkarainen A., Lundbom J., Vuolteenaho K., Saarinen L., Hautaniemi S., Rodriguez A., Frühbeck G., Pajunen P. (2014). Characterising metabolically healthy obesity in weight-discordant monozygotic twins. Diabetologia.

[64] Defour M., Michielsen C.C.J.R., O’Donovan S.D., Afman L.A., Kersten S. (2020). Transcriptomic signature of fasting in human adipose tissue. Physiol. Genomics.

[65] Johansson L.E., Danielsson A.P.H., Parikh H., Klintenberg M., Norström F., Groop L., Ridderstråle M. (2012). Differential gene expression in adipose tissue from obese human subjects during weight loss and weight maintenance. Am. J. Clin. Nutr..

[66] Matualatupauw J.C., Bohl M., Gregersen S., Hermansen K., Afman L.A. (2017). Dietary medium-chain saturated fatty acids induce gene expression of energy metabolism-related pathways in adipose tissue of abdominally obese subjects. Int. J. Obes..

[67] du Plessis J., van Pelt J., Korf H., Mathieu C., van der Schueren B., Lannoo M., Oyen T., Topal B., Fetter G., Nayler S. (2015). Association of Adipose Tissue Inflammation With Histologic Severity of Nonalcoholic Fatty Liver Disease. Gastroenterology.

[68] Van Bussel I.P.G., Backx E.M.P., De Groot C.P.G.M., Tieland M., Müller M., Afman L.A. (2017). The impact of protein quantity during energy restriction on genome-wide gene expression in adipose tissue of obese humans. Int. J. Obes..

[69] MacLaren R.E., Cui W., Lu H., Simard S., Cianflone K. (2010). Association of adipocyte genes with ASP expression: a microarray analysis of subcutaneous and omental adipose tissue in morbidly obese subjects. BMC Med. Genomics.

[70] Hardy O.T., Perugini R.A., Nicoloro S.M., Gallagher-Dorval K., Puri V., Straubhaar J., Czech M.P. (2011). Body mass index-independent inflammation in omental adipose tissue associated with insulin resistance in morbid obesity. Surg. Obes. Relat. Dis. Off. J. Am. Soc. Bariatr. Surg..

[71] Salcedo-Tacuma D., Bonilla L., Montes M.C.G., Gonzalez J.E.N., Gutierrez S.M.S., Chirivi M., Contreras G.A. (2022). Transcriptome dataset of omental and subcutaneous adipose tissues from gestational diabetes patients. Sci. Data.

[72] Aguilera C.M., Gomez-Llorente C., Tofe I., Gil-Campos M., Cañete R., Gil Á. (2015). Genome-wide expression in visceral adipose tissue from obese prepubertal children. Int. J. Mol. Sci..

[73] Grundberg E., Small K.S., Hedman Å.K., Nica A.C., Buil A., Keildson S., Bell J.T., Yang T.-P., Meduri E., Barrett A. (2012). Mapping cis- and trans-regulatory effects across multiple tissues in twins. Nat. Genet..

[74] Heinonen S., Muniandy M., Buzkova J., Mardinoglu A., Rodríguez A., Frühbeck G., Hakkarainen A., Lundbom J., Lundbom N., Kaprio J. (2017). Mitochondria-related transcriptional signature is downregulated in adipocytes in obesity: a study of young healthy MZ twins. Diabetologia.

[75] Bollepalli, S., Kaye, S., Heinonen, S., Kaprio, J., Rissanen, A., Virtanen, K.A., Pietiläinen, K.H., and Ollikainen, M. (2018). Subcutaneous adipose tissue gene expression and DNA methylation respond to both short- and long-term weight loss. Int. J. Obes. 2005 *42*, 412–423. 10.1038/ijo.2017.245. 28978976

[76] Rey F., Messa L., Pandini C., Launi R., Barzaghini B., Micheletto G., Raimondi M.T., Bertoli S., Cereda C., Zuccotti G.V. (2021). Transcriptome Analysis of Subcutaneous Adipose Tissue from Severely Obese Patients Highlights Deregulation Profiles in Coding and Non-Coding Oncogenes. Int. J. Mol. Sci..

[77] Diamanti K., Cavalli M., Pereira M.J., Pan G., Castillejo-López C., Kumar C., Mundt F., Komorowski J., Deshmukh A.S., Mann M. (2022). Organ-specific metabolic pathways distinguish prediabetes, type 2 diabetes, and normal tissues. Cell Rep. Med..

[78] Hruska P., Kucera J., Kuruczova D., Buzga M., Pekar M., Holeczy P., Potesil D., Zdrahal Z., Bienertova-Vasku J. (2023). Unraveling adipose tissue proteomic landscapes in severe obesity: insights into metabolic complications and potential biomarkers. Am. J. Physiol. Endocrinol. Metab..

[79] Rydén M., Andersson D.P., Kotopouli M.I., Stenberg E., Näslund E., Thorell A., Sørensen T.I.A., Arner P. (2022). Lipolysis defect in people with obesity who undergo metabolic surgery. J. Intern. Med..

[80] Rydén M., Hrydziuszko O., Mileti E., Raman A., Bornholdt J., Boyd M., Toft E., Qvist V., Näslund E., Thorell A. (2016). The Adipose Transcriptional Response to Insulin Is Determined by Obesity, Not Insulin Sensitivity. Cell Rep..

[81] Arner P., Rydén M. (2015). Fatty Acids, Obesity and Insulin Resistance. Obes. Facts.

[82] Andersson D.P., Kerr A.G., Dahlman I., Rydén M., Arner P. (2023). Relationship Between a Sedentary Lifestyle and Adipose Insulin Resistance. Diabetes.

[83] Larsen T.M., Dalskov S.-M., van Baak M., Jebb S.A., Papadaki A., Pfeiffer A.F.H., Martinez J.A., Handjieva-Darlenska T., Kunešová M., Pihlsgård M. (2010). Diets with high or low protein content and glycemic index for weight-loss maintenance. N. Engl. J. Med..

[84] Kerr A.G., Andersson D.P., Rydén M., Arner P. (2024). Insulin resistance in adipocytes: Novel insights into the pathophysiology of metabolic syndrome. Clin. Nutr. Edinb. Scotl..

[85] Arner P., Bolinder J., Hellmér J., Engfeldt P. (1986). Methods in Diabetes Research.

[86] Löfgren P., Hoffstedt J., Rydén M., Thörne A., Holm C., Wahrenberg H., Arner P. (2002). Major gender differences in the lipolytic capacity of abdominal subcutaneous fat cells in obesity observed before and after long-term weight reduction. J. Clin. Endocrinol. Metab..

[87] Arner P., Hellmér J., Wennlund A., Ostman J., Engfeldt P. (1988). Adrenoceptor occupancy in isolated human fat cells and its relationship with lipolysis rate. Eur. J. Pharmacol..

[88] Kenakin T.P. (1984). The classification of drugs and drug receptors in isolated tissues. Pharmacol. Rev..

[89] Boone-Villa D., Ventura-Sobrevilla J., Aguilera-Méndez A., Jiménez-Villarreal J. (2022). The effect of adenosine monophosphate-activated protein kinase on lipolysis in adipose tissue: an historical and comprehensive review. Arch. Physiol. Biochem..

[90] Collins S. (2022). β-Adrenergic Receptors and Adipose Tissue Metabolism: Evolution of an Old Story. Annu. Rev. Physiol..

[91] Cotecchia S., Stanasila L., Diviani D. (2012). Protein-protein interactions at the adrenergic receptors. Curr. Drug Targets.

[92] Syrovatkina V., Alegre K.O., Dey R., Huang X.-Y. (2016). Regulation, Signaling, and Physiological Functions of G-Proteins. J. Mol. Biol..

[93] Yu H.C., Jeon Y.G., Na A.-Y., Han C.Y., Lee M.R., Yang J.D., Yu H.C., Son J.B., Kim N.D., Kim J.B. (2024). p21-activated kinase 4 counteracts PKA-dependent lipolysis by phosphorylating FABP4 and HSL. Nat. Metab..

[94] Dobin A., Davis C.A., Schlesinger F., Drenkow J., Zaleski C., Jha S., Batut P., Chaisson M., Gingeras T.R. (2013). STAR: ultrafast universal RNA-seq aligner. Bioinforma. Oxf. Engl..

[95] Lawrence M., Huber W., Pagès H., Aboyoun P., Carlson M., Gentleman R., Morgan M.T., Carey V.J. (2013). Software for Computing and Annotating Genomic Ranges. PLoS Comput. Biol..

[96] Cacciamani G.E., Chu T.N., Sanford D.I., Abreu A., Duddalwar V., Oberai A., Kuo C.-C.J., Liu X., Denniston A.K., Vasey B. (2023). PRISMA AI reporting guidelines for systematic reviews and meta-analyses on AI in healthcare. Nat. Med..

