# Supplementary material for: The nature of sex differences in catecholamine-induced lipolysis in subcutaneous fat cells
Source: iScience. 2025 Nov 10;28(12):113988. doi: 10.1016/j.isci.2025.113988 (PMC12765390; doi:10.1016/j.isci.2025.113988)
Supplement: Document S1. Tables S1–S5 [file mmc1.pdf]

iScience, Volume *28*

Supplemental information

The nature of sex differences

in catecholamine-induced lipolysis

in subcutaneous fat cells

Lucas Massier, Daniel P. Andersson, Nathalie Viguerie, Jiawei Zhong, Danae Zareifi, Alastair G. Kerr, Dominique Langin, and Peter Arner

Table S1. Clinical characteristics of men and women investigated for lipolysis. Values are mean and (range) and were compared by unpaired t-test or Fisher´s exact test. n = number of subjects. Obesity was defined as body mass index ≥30 kg/m^2.

Phenotype	Women		Men		p-value
	Mean and (range)	n	Mean and (range)	n	
Age years)	40 (18-79)	773	46 (18-79)	300	<0.0001
Waist to hip (ratio)	0.92 (0.76-1.17)	758	0.99 (0.80-1.21)	279	<0.0001
Body mass index (kg/m^2)	34 (15-63)	774	30 (19-53)	298	<0.0001
Body fat content by impedance (%)	50 (22.-75)	623	28 (8-63)	223	<0.0001
S-insulin (mU/l)	12 (1-58)	745	13 (2-68)	289	0.10
P-glucose (mmol/l)	5.4 (3.4-20.9)	765	5.8(3.6-18.4)	277	<0.0001
P-triglycerides (mmol/l)	1.4 (0.0-7.9)	750	2.0 (0.3-21.7)	295	<0.0001
P-total cholesterol (mmol/l)	5.0 (2.5-12.2)	754	5.3 (2.5-12.8)	294	0.001
P-HDL cholesterol (mmol/l)	1.3 (0.5-2.9)	727	1.2 (0.5-2.3)	289	<0.0001
Diastolic blood pressure (mm Hg)	77 (45-119)	709	81 58-125)	265	<0.0001
Systolic blood pressure (mm Hg)	127 (84-182)	713	134 (101-195)	264	<0.0001
Fat cell volume (picolitres)	718 (92-1452)	766	603 (72-1375)	288	<0.0001
Nicotine use (yes/no)	190/566	756	90/198	288	0.051
Sedentary/physically active lifestyle	231/427	658	64/146	210	0.24
Obesity (yes/no)	491/283	774	197/101	298	<0.0001
Cardiometabolic disease (yes/no)	112/662	774	96/204	300	<0.0001

Table S2. Clinical characteristics of men and women investigated for RNAseq in the DiOGenes group. Values are mean and (range) and were compared by unpaired t-test or Fisher´s exact test. n = number of subjects. Obesity was defined as body mass index ≥30 kg/m^2.

Phenotype	Women		Men		p-value
	Mean and (range)	n	Mean and (range)	n	
Age years)	41 (24-56)	276	43 (28-63)	125	0.009
Body mass index (kg/m^2)	35 (26-48)	272	34 (26-47)	123	0.39
Body fat content by impedance (%)	44 (32.-62)	235	33 (21-68)	109	<0.0001
S-insulin (mU/l)	11 (2-156)	257	14 (3-138)	119	0.018
P-glucose (mmol/l)	5.0 (2.9-8.5)	258	5.3 (3.4-7.3)	118	0.002
P-triglycerides (mmol/l)	1.2 (0.4-3.8)	268	1.6 (0.4-3.8)	119	<0.0001
P-total cholesterol (mmol/l)	4.9 (2.3-7.7)	272	5.2 (1.7-8.4)	121	0.004
P-HDL cholesterol (mmol/l)	1.3 (0.5-2.5)	272	1.1 (0.5-2.4)	121	<0.0001
Obesity (yes/no)	230/43	273	103/20	123	0.88

Table S3. Influence of cofactors on the impact of sex on noradrenaline induced lipolysis expressed as (10)log noradrenaline/basal glycerol release. ANCOVA was used in the four different models putting sex together with either body mass index, body fat content, age, or waist to hip ratio.

Condition	F-value	p-value
Sex (758 women and 282 men)	71	<0.0001
Body mass index (kg/m^2)	48	<0.0001
Sex X body mass index	2.5	0.11
Sex (613 women and 220 men)	66	<0.0001
Body fat by impedance (%)	32	<0.0001
Sex X body fat	0.8	0.38
Sex (757 women and 283 men)	31	<0.0001
Age (years)	47	<0.0001
Sex X age	1.2	0.27
Sex (745 women and 274 men)	18	<0.0001
Waist to hip (ratio)	43	<0.0001
Sex X waist to hip	3.1	0.08

Table S4 List of genes involved in fat cell lipolysis stimulation by catecholamines through the canonical cyclic AMP pathway and additional genes associated with this pathway of lipolysis regulation. References are found in the Methods section.

Genes	Role in lipolysis
**ADRENERGIC RECEPTORS**<br>*ADRB1*encoding beta-1 adrenergic receptor<br>*ADRB2* encoding beta-2 adrenergic receptor<br>*ASDRB3* encoding beta-3 adrenergic receptor<br>*ADR2A* encoding alpha-2A adrenergic receptor	The beta receptors stimulate lipolysis. The alpha-2A receptor inhibits lipolysis.
**G-PROTEINS**<br>*GNAI1* encoding Gi-1 alpha protein<br>*GNAI2* encoding Gi-2 alpha protein<br>*GNAI3* encoding Gi-2 alpha protein<br>*GNAO1* encoding Go-1 alpha protein<br>*GNAZ* encoding Gz protein<br>*GNAT1* encoding Gt-1 alpha protein<br>*GNAT2* encoding Gt-2 alpha protein<br>*GNAT3* encoding Gt-3 alpha protein<br>*GNAB3* encoding Gs beta-3 subunit protein<br>*GNAS* encoding Gs alpha subunit protein	Couple adrenoceptors in a positive (for the beta-subtypes) or a negative (for the alpha-2A subtype) fashion to adenylyl cyclase.
**ADENYLYL CYCLASE**<br>*ADCY3* encoding adenylyl cyclase 3<br>*ADCY5* encoding adenylyl cyclase 5	Stimulates formation of cyclic adenosine monophosphate (cAMP).
**PROTEIN KINASE A (PKA) COMPLEX**<br>*PRKACA* encoding protein kinase cAMP activated catalytic subunit alpha<br>*PRKACB* encoding protein kinase cAMP activated catalytic subunit beta<br>*PRKACG* encoding protein kinase cAMP activated catalytic subunit gamma<br>*PRKAR1A* encoding protein kinase cAMP dependent type 1 regulatory subunit alpha<br>*PRKAR1B* encoding protein kinase cAMP dependent type 1 regulatory subunit beta<br>*PRKAR2A* encoding protein kinase cAMP dependent type 2 regulatory subunit alpha<br>*PRKAR2B* encoding protein kinase cAMP dependent type 2 regulatory subunit beta	Activate lipases after stimulation by cAMP
**LIPASES**<br>*MGLL* encoding monoacylglycerol lipase<br>*LIPE* encoding hormone sensitive lipase<br>*PNPLA2* encoding adipose triglyceride lipase	Stimulate the stepwise hydrolysis (lipolysis) of triacylglycerols to fatty acids and glycerol.
**BETA ARRESTINS**<br>*ARRB1* encoding arrestin beta-1<br>*ARRB2* encoding arrestin beta-2	Cause desensitization of adrenoceptors and diminish their action on lipolysis.
**ADENOSINE MONOPHOSPHATE ACTIVATED PROTEIN KINASE COMPLEX**<br>*PRKAA1* encoding 5'-AMP activated protein kinase catalytic subunit alpha-1<br>*PRKAA2* encoding 5'-AMP activated protein kinase catalytic subunit alpha-2	Activated by catecholamine stimulation and inhibit lipases causing a break on lipolysis.

PRKAB1 encoding 5'-AMP activated protein kinase non-regulatory subunit beta-1<br>*PRKAB2* encoding 5'-AMP activated protein kinase non-regulatory subunit beta-2<br>PRKAG1 encoding 5'-AMP activated protein kinase non-regulatory subunit gamma-1<br>*PRKAG2* encoding 5'-AMP activated protein kinase non-regulatory subunit gamma-2<br>*PRKAG3* encoding 5'-AMP activated protein kinase non-regulatory subunit gamma-3	
**ADDITIONAL GENES**	
PLIN1 encoding perilipin-1	Activates HSL
GOS2 encoding GO/G1 Switch 2	Lipase inhibitor
ABHD5 encoding abhydrolase domain containing 5' lysophosphatidic acid acyltransferase (CGI-58)	Cofactor for adipose triglyceride lipase
PDE3B encoding phosphodiesterase 3b	Stimulates degradation of cAMP to inactive 5' AMP
CIDEA encoding cell death inducing DFFA like effector A	Inhibits basal and stimulated lipolysis
CIDEC encoding cell death inducing DFFA like effector C	Modulates lipolysis through interaction with CGI-58
FABP4 encoding fatty acid binding protein 4	Binds to intracellular fatty acids and HSL participating in lipolysis activation.
CAVIN1 (PRTF) encoding caveolae associated protein 1	Binds to HSL and modifies stimulated lipolysis

Table S5: Datasets included in transcriptome and proteome analyses.

Publication	Year	PMID	Data source	Module
Transcriptome				
Kerr, AG. (2020)	2020	32406570	GSE199063	Clinical
Imbert, A. (2022)	2022	34415992	GSE141221	Clinical
Armenise, C. (2017)	2017	28793995	GSE95640	Clinical
Arner, P. (2016)	2016	27535281	GSE76399	Clinical
Krieg, L. (2021)	2021	34598978	Manuscript files	Clinical
Keller, M. (2017)	2017	28123940	communication with author	Clinical
Petrus, P. (2018)	2018	30332637	GSE59034	Clinical
Arner, E. (2012)	2012	22688341	GSE25402	Clinical
Arner, P. (2018)	2018	29861390	GSE113080	Clinical
Nono Nankam, PA. (2020)	2020	32581226	communication with author	Clinical
Civelek, M. (2017)	2017	28257690	GSE70353	Clinical
Raulerson, C. (2019)	2019	31564431	GSE135134	Clinical
Stančáková, A. (2012)	2012	22553379	GSE32512	Clinical
Winnier, DA. (2015)	2015	25830378	GSE64567	Clinical
Nookaew, I. (2013)	2013	23264395	GSE27916	Clinical
Das, SK. (2015)	2015	25868721	GSE65221	Clinical
Barberio, MD. (2019)	2019	31798691	GSE88837	Clinical
Vink, RG. (2017)	2017	27840413	GSE77962	Clinical
Sharma, NK. (2016)	2016	26789776	GSE95674	Clinical
GTEx microarray	2013		GSE45878	Clinical
Lonsdale, J. (2013)	2013	23715323	gtexportal	Clinical

Aguet, F. (2017)	2017	29022597	gtexportal	Clinical
GTEx v7			gtexportal	Clinical
Aguet, F. (2020)	2020	32913098	gtexportal v8	Clinical
Drong, A. (2013)	2013	23431366	E-MTAB-54	Clinical
Naukkarinen, J. (2013)	2013	24100782	E-MTAB-1895	Clinical
Defour, M. (2020)	2020	32866087	GSE154610	Clinical
Johansson, LE. (2012)	2012	22648723	GSE35411	Clinical
Matualatupauw, JC. (2017)	2017	28529330	GSE87382	Clinical
Du Plessis, J. (2015)	2015	26028579	GSE58979	Clinical
Van Bussel, IPG. (2017)	2017	28337029	GSE84046	Clinical
MacLaren, RE. (2010)	2010	20105310	GSE15524	Clinical
Hardy, OT. (2011)	2011	20678967	GSE20950	Clinical
Salcedo-Tacuma, D. (2022)	2022	35715414	GSE188799	Clinical
Aguilera, CM. (2015)	2015	25856673	GSE9624	Clinical
Grundberg, E. (2012)	2012	22941192	E-TABM-1140	Clinical
Heinonen, S. (2017)	2017	27734103	GSE92405	Clinical
Bollepalli, S. (2018)	2018	28978976	GSE103766	Clinical
Rey, F. (2021)	2021	33671464	GSE166047	Clinical
Diamanti, K. (2022)	2022	36198307	PXD027597	Clinical
Proteome				
Zhong et al.	2025	39983713	PXD057443	Clinical
Hruska, P. (2023)	2023	37792298	PXD041721	Clinical
Krieg, L. (2021)	2021	34598978	Manuscript files	Clinical
